# Influences of Language Functions on Linguistic Features: Multi-Dimensional and Entropy Analyses of Academic and Entertainment Registers

**DOI:** 10.3390/e27080783

**Published:** 2025-07-24

**Authors:** Changwei Hu, Yu Zhu, Liangjie Yuan

**Affiliations:** 1 International Cultural and Educational College, Northeast Agricultural University, Harbin 150030, China; hucw@neau.edu.cn; 2 Chinese International Education College, Xiamen University, Xiamen 361102, China; zhuyu@xmu.edu.cn; 3 Department of Chinese Language and Literature, Xiamen University, Xiamen 361005, China

**Keywords:** linguistic features, language function, multi-dimensional analysis, entropy, register variation

## Abstract

This study examines how language functions impact linguistic features in academic and entertainment registers. Using multi-dimensional analysis (MDA) and computing entropy values, we analyze a large-scale Chinese corpus consisting of over 19 million tokens from 1000 texts, including academic journals, dissertations, entertainment magazines, and novellas. We identify key language functions that shape linguistic features within these registers. Our results reveal five core dimensions of linguistic functional variation, narrative versus rational discourse, modification, reference, uncertainty, and prudence, which account for over 52% of the variance in language use. Certain linguistic features systematically co-occur in each dimension, forming language functions that underpin broader social networks. Entropy values further confirm the findings of multi-dimensional analysis. This study emphasizes the associations between linguistic features and language functions, offering a theoretical perspective for understanding how language functions impact linguistic features and shape different registers. The findings suggest a language variation perspective on social networks’ communication.

## 1. Introduction

Language serves as a fundamental tool for human communication, with its usage closely tied to the various functions it fulfills in social contexts. Online and offline social networks significantly influence how language is structured and used in different settings. The relationship between language and social networks has been a topic of interest in sociolinguistics and communication studies for decades [1,2,3]. One important aspect of language within social networks is how linguistic features signal different roles or positions within a network [4,5]. Numerous studies have explored how language features operate in social networks and contribute to the formation of communities. For instance, research on discourse and social networks has demonstrated that language can reveal information flow within these networks and the dynamics of power relations [6,7]. Similarly, language functions transcend mere information transmission and vary with social context [8].

In general terms, a register refers to a variety of language associated with a particular situation, including specific communicative purposes [9,10]. Registers are typically described by their characteristic uses of lexical and grammatical features, which vary according to the context in which they are employed. For example, academic language and entertainment language are distinct registers that serve different social functions—academic language prioritizes precision, objectivity, and intellectual rigor; entertainment language is geared toward engaging, entertaining, and appealing to emotions. These contrasting language functions shape their respective uses of linguistic features, such as vocabulary, sentence patterns, and tone, aligning with each register’s broader communicative goals.

In both academic and entertainment contexts, language serves as a tool for navigating social situations, where specific linguistic choices can align with professional or casual identities, facilitating the construction and negotiation of social relationships.

This topic has been explored in studies on academic English, such as those by Biber [11], who investigated language variation in American higher education along two dimensions: speech versus writing and academic versus nonacademic language. Moreover, research by Nesi and Gardner [12] examined language variation in academic writing across grades, various subjects, and genres, offering key insights into the use of academic language. However, studies on language variation in non-English academic settings, particularly in languages such as Chinese, remain underdeveloped [13].

The development of linguistic analysis methods has made it possible to examine language across various dimensions, offering deeper insights into how the functions influence its features. A key methodology for exploring language variation is multi-dimensional analysis (MDA), a quantitative method developed by Biber [14,15,16] that enables researchers to analyze the complexity of language across various registers. MDA identifies patterns of linguistic features that differentiate registers or communication contexts by considering multiple dimensions simultaneously, such as syntactic complexity, lexical diversity, and sentence structure [11,17]. In recent years, MDA has been widely applied to academic English to examine register variation. Studies by Biber et al. [18], for example, have focused on grammatical complexity in English for Academic Purposes (EAP), while other research has investigated variations in academic writing across different sub-registers, including personal versus objective genres [19] and native versus non-native learners [20,21]. Other studies have explored professional academic writing [22,23,24,25] and even the interaction between different research reports and disciplines, as exemplified by Gray [26].

These studies confirm the existence of key dimensions of language variation, such as ‘involved vs. informational’ and ‘narrative vs. non-narrative,’ which are present across various forms of academic writing [11]. Additionally, ‘stance’ and ‘persuasion’ have been identified as significant dimensions of language variation in academic discourse; however, the specific terms used in different studies are not always consistent. For instance, ‘stance’ has been referred to as ‘personal opinion’ [20], ‘expressions of opinions, attitudes, emotions, and mental processes’ [19,21,27], and ‘author-centered stance’ [24]. Similarly, ‘persuasion’ is described as ‘reflective/argumentative discourse’ [20], ‘production of possibility and argumentation’ [19,21,27], ‘overt expression of persuasion’ [16,22], and ‘explicit versus implicit argumentation’ [23].

In addition to academic discourse, MDA has been utilized to analyze language variation in various domains, including fiction [28], internet texts [29,30,31], and television programs [32]. These studies have revealed common dimensions, such as ‘involved vs. informational’, and unique dimensions specific to each domain, such as ‘exposition or discussion vs. simplified interaction’ in television programs [20]. However, despite the growing application of MDA across different registers, there is a need for further studies, particularly in non-English contexts, which investigate the comparative patterns of language variation between academic and non-academic registers—an area that holds promise for enhancing our understanding of the universal language dimensions proposed by Biber [17].

Additionally, entropy, an important quantitative measure used to assess the information content of texts, was first defined by Shannon [33] and has since been widely applied in numerous studies. Entropy allows for the calculation of information content of a given text sample, and in a simple way, information can be parameterized by word or part-of-speech diversity [34]. For example, Christian et al. [35] studied over 1000 languages and discovered that word entropies exhibit relatively narrow, unimodal distributions, aligning with information-theoretic communication models. Other research has found that entropies are sensitive to language and text categories [36,37]. These findings reveal patterns inherent in entropy values as a linguistic feature, which can indicate group membership and social identity, thereby influencing the creation and maintenance of network communities. It should be specially noted that entropy reflects unpredictability, not necessarily structural or functional complexity. For instance, a randomly generated text could have high entropy but no communicative value; thus, it possesses null complexity. Thus, the present study uses real natural language as the corpus, excluding randomly generated meaningless content, and utilizes entropy value calculations to discuss and supplement language functions such as “uncertainty.”

To summarize, this study will employ MDA and entropy analysis on a substantial corpus of both academic and entertainment Chinese texts to examine how language functions impact distinct linguistic features and influence broader social network communications in these contexts. The findings will enhance our understanding of how language functions across various registers, deepening our comprehension of the relationship between language variation and social contexts. Through this approach, the research aims to provide new theoretical insights into how language functions influence and are influenced by interactions within academic and entertainment registers.

The primary contributions of this study are outlined as follows:

- This paper explores the influence of language functions on linguistic features. It identifies five key dimensions of linguistic variation: narrative versus rational discourse, modification, reference, uncertainty, and prudence.
- By analyzing a comprehensive Chinese corpus, this study offers a theoretical perspective for understanding how language functions impact linguistic features and shape different registers.
- For the language functions of “uncertainty,” this paper explains and illustrates them through entropy value calculation, which confirms the findings of multidimensional analysis and reflects unpredictability of language use in specific registers.

The remainder of this article is organized as follows: Section 2 presents the research questions, the corpus under analysis, and a thorough explanation of the MDA framework, which includes linguistic features as well as the instruments and procedures utilized, followed by the methods for calculating entropy. Section 3 provides the outcomes of the multi-dimensional analysis encompassing five key dimensions, along with the entropy calculation results across character, word, and sentence lengths. Section 4 discusses the results in both academic and entertainment registers. Finally, Section 5 summarizes the findings and contributions of this study and proposes directions for future work.

## 2. Materials and Methods

### 2.1. Research Questions

More specifically, the questions to be addressed in this study are as follows:

1. How do language functions influence the use of linguistic features in academic registers (such as journal papers and research dissertations) and entertainment registers (like popular novellas and magazine articles)? How do entropy values vary across the two registers, and how are they influenced by language functions?
2. In what ways do linguistic features within the academic and entertainment registers vary, and how do these variations contribute to distinct language functions that shape communication in these contexts? How do entropy values vary within the two registers, and how are they influenced by language functions?

### 2.2. The Corpus Under Analysis

The corpus constructed for this study comprises 1000 texts, totaling over 19,000,000 characters. The academic section includes 300 academic papers from 28 leading Chinese journals (3,250,000 characters) and 200 research dissertations from top-ranking Chinese universities (6,550,000 characters). The entertainment section features 300 randomly selected articles from popular entertainment magazines (3,010,000 characters) and 200 widely read novellas (6,590,000 characters). All the texts for this corpus were sourced from publicly accessible internet platforms, including the CNKI academic database and the websites of entertainment magazines and novella publishers.

This study employs complete texts rather than segmented language fragments in the corpus to reflect authentic language contexts more accurately. Since dissertations and novellas are typically much longer than journal papers and magazine articles, the number of texts in each category differs. However, the overall character count in both the academic and entertainment categories remains largely consistent.

### 2.3. Multi-Dimensional Analysis Framework

#### 2.3.1. Linguistic Features

This study analyzes 97 linguistic features listed in Appendix A. These features aim to cover the important elements identified in previous research comprehensively.

Out of these, 47 features identified by Biber [16] apply to both English and Chinese and are presented without any superscripts. Additionally, 23 features from Zhu [13] are marked with a superscript “a,” as they primarily reflect unique characteristics of Chinese. Examples include the particles 着 (*zhe*), 了 (*le*), 过 (*guò*), 的 (*de*), 地 (*de*), and 得 (*de*).

Furthermore, three features that denote the most frequently used nouns, verbs, and adjectives—absent in Biber [16]—are included as candidates, marked with a superscript “b.” The analysis also incorporates the 10 types of compound sentences discussed by Huang and Liao [38], labeled with a superscript “c”.

Then, 11 linguistic features labeled with a superscript “d” are included because they are regarded as distinguishing traits between academic and non-academic written Chinese, according to the previous literature. For instance, the indexes of formality in Chinese texts, such as 嵌偶单音词 (*qiàn ǒu dān yīn cí*), meaning “monosyllabic words used in disyllabic templates,” and 古语词 (*gǔ yǔ cí*), meaning “classical Chinese words,” are cited by Feng et al. [39]. Similarly, 口语词 (*kǒu yǔ cí*), meaning “oral words,” and 儿化词 (*ér huà cí*), meaning “-er words,” as noted by Li [40], are also included.

Lastly, three commonly reported items in corpus linguistic studies—the type–token ratio, average word length, and average sentence length—are denoted with a superscript “e” and included as linguistic features in this study.

#### 2.3.2. Instruments and Procedure

The main instruments used in this analysis include the following:

- The NLPIR system (developed by the Institute of Computing Technology, Chinese Academy of Sciences) for parsing and tagging the texts in the corpus.
- AntConc for concordancing.
- A self-developed program called “Text_Analysis” for additional concordancing.

The procedures for MDA are as follows:

1. Parsing, tagging, and concordancing using both software and manual proofreading.
2. Counting the frequency of linguistic features.
3. Normalizing the frequencies of these linguistic features.
4. Conducting factor analysis to explore the co-occurrence patterns of linguistic features.
5. Calculating factor scores.
6. Comparing factor scores and discussing variations in language.

The method used to calculate factor scores is the regression method, which applies the following general formula based on standardized (*z*-scored) observed variables:

(1) $$F_{j}\;=\sum_{i=1}^{p}w_{ij}\cdot z_{i}$$

where

*F_j_*: estimated factor score for factor *j*;

*w_ij_*: factor score coefficient (weight) for variable *i* on factor *j*;

*z_i_*: standardized (*z*-score) value of observed variable *i*;

*p*: number of observed variables.

Additionally, the method for computing the weights *w_ij_* follows the regression approach, also referred to as Bartlett’s method. The formula for the weights is derived as follows:

(2) $$W=L^{T}\Psi^{-1}{\left(L\Psi^{-1}L^{T}\right)}^{-1}$$

where

**W**: matrix of regression weights (used to compute factor scores);

**L**: matrix of factor loadings;

**Ψ**: diagonal matrix of unique variances (i.e., uniquenesses of each variable).

This formula ensures that the factor scores are linear combinations of observed variables, providing best linear unbiased estimates (BLUE) of the factor scores under the assumptions of the factor model.

### 2.4. Entropy Calculation Methods

In this study, we utilize methods from information theory [41] that are based on entropy to quantify the complexity of language in the text. Specifically, we analyze character-level entropy, word-level entropy, and sentence-length entropy. These metrics allow us to evaluate the uncertainty and information density of the text, providing insights into its linguistic complexity. The entropy *H* is mathematically defined as

(3) $$H=-\sum_{i}p_{i}\log_{2}p_{i}$$

where *p_i_* represents the probability of occurrence for a given unit (character, word, or sentence length).

1. Character-level entropy quantifies the uncertainty or average information content of characters in the text. It is estimated by calculating the frequency distribution of characters within the text.
2. Word-level entropy measures the uncertainty in the selection of words from a given vocabulary. The calculation of word-level entropy must also account for dependencies between words, as word occurrences are context dependent in natural language.
3. Sentence-length entropy captures the complexity arising from variations in sentence lengths within the text. This is determined by analyzing the frequency distribution of sentence lengths (in terms of the number of words) in the text, allowing for an evaluation of structural complexity.

By calculating these three measures of entropy, we can perform a thorough analysis of the linguistic complexity found in the text. These entropy values provide a quantitative framework for understanding the structural and informational features inherent in the language used within the text.

## 3. Results

### 3.1. Multi-Dimensional Analysis Results

**Figure 1:**
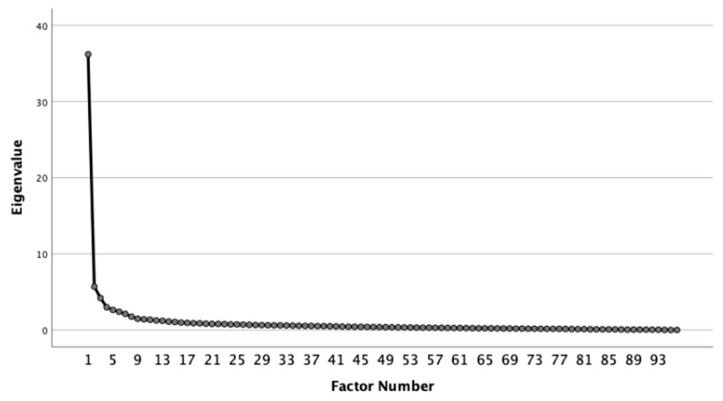
The scree plot of the present study.

The common factor analysis of the data from this study was conducted using IBM SPSS Statistics 26.0. The analysis was supported by a high KMO value of 0.97 and a low *p*-value for the Bartlett’s sphericity test (*p* = 0.000). The Unweighted Least Squares method was employed for factor extraction, and Promax was used for factor rotation; the resulting scree plot is shown in Figure 1.

Eigenvalues stabilize after the fifth factor. The first five factors explain more than 52% of the total variance, highlighting the main dimensions of language variation among the texts in this study’s corpus and the networks of language structures, each of which will be discussed next.

#### 3.1.1. Dimension 1: Narrative Discourse vs. Rational Discourse

The first dimension includes 56 linguistic features (see Appendix B.1 for details) and accounts for 35.45% of the total variance in the data. The 40 features with positive loadings are linked to producing narrative discourses in an engaged style. It emphasizes pronouns (second person, first person, third person, indefinite, demonstrative), verbs serving various functions (directional, communicative, speculative, mental), modals (modal particle, possibility modal), and tenses (着 *zhe* ’past aspect, progressive construal’). Moreover, auxiliary features (e.g., 得 *de*, 地 *de*) and adverbial features (obligatory adverb, adverb of time, attitude adverb, frequently used adverb, locative words) closely related to verbs in narrative discourses are included in the positive group. Common expressions in spoken Chinese (e.g., onomatopoeia, interjections, spoken word) also play a significant role in the positive group, contributing to a casual, everyday conversation style.

The negative group in this dimension consists of 10 features commonly observed in written academic discourse. Among these, nouns are highly concentrated (noun: most commonly used, abstract noun), reflecting that academic texts often engage with concepts, particularly abstract ones. Verbs in the negative group (gerundive functional word, light verb) tend to be more abstract or nominalized. Explanatory compound sentences provide detailed explanations for complex issues typically encountered in academic contexts. Coupled disyllabic words imply a strong sense of formal discourse and are rarely found in informal settings. Average word length, average sentence length, and type–token ratio are widely recognized as differing between academic and entertainment texts, with the former usually featuring longer words, longer sentences, and a higher type–token ratio. The analogous particle “等 *děng*/等等 *děng děng*”, equivalent to “and so on” or “etc.,” is also frequently used in expository texts, especially when providing examples. The remaining two features in this group (i.e., distinguishing words, metrics & measures) can be utilized to differentiate or define concepts.

#### 3.1.2. Dimension 2: Modification

Dimension 2 comprises five linguistic features (see Appendix B.2 for details), accounting for 5.55% of the total variance. Among these features, moderately to rarely used adverbs and adjectives are typically used to modify verbs and nouns, respectively. Additionally, a monosyllabic word within a disyllabic template is considered an index of the degree of gracefulness in Chinese texts [39]. Therefore, the function of this dimension is ‘modification’.

#### 3.1.3. Dimension 3: Reference

The frequent co-occurrence of six specific features—namely, mental nouns, the inanimate pronoun 它 (*tā*) meaning ‘it’, the particle 的 (*de*), the copular verb 是 (*shì*) which means ‘to be’ and serves as the main verb, the first-person pronoun 我们 (*wǒmen*) meaning ‘we’, and university subject classification—often appears in academic texts to fulfill a referencing function. Therefore, this aspect is designated as ‘reference’.

#### 3.1.4. Dimension 4: Uncertainty

The fourth co-occurrence pattern includes two linguistic features: adverbial hedges and possibility adverbs, which together account for 2.35% of the total variance. This pattern is referred to as ‘uncertainty’.

#### 3.1.5. Dimension 5: Prudence

The last dimension consists of four linguistic features: numerals, sequential words, classical words, and metrics and measures. Both numerals and metrics and measures are essential for defining quantities. Classical words effectively indicate the level of formality in written Chinese [39]. Sequential words enhance cohesion and coherence at the textual level, helping to organize the ideas presented in the text. A logically organized expression contributes to a sense of prudence in the text. Therefore, this dimension is referred to as ‘prudence’.

### 3.2. Register Variations Across Academic and Entertainment

After establishing linguistic dimensions through MDA, factor scores from regression analysis using SPSS software provided a quantitative assessment of the linguistic features involved. These factor scores reveal the underlying patterns of language use within each text type and serve as multivariates in subsequent analyses. MANOVA was used to examine differences in the five dimensions of linguistic variation across registers, confirming that register type significantly influenced these dimensions.

Initially, twenty multivariate outliers were identified and removed, leaving 980 texts available for further analysis.

Secondly, the Box’s M test indicated a violation of the homogeneity assumption, with a *p*-value of 0.000. As noted by [42], Pillai’s trace is considered the most powerful and robust method, making it the preferred choice for conducting MANOVA when the homogeneity of covariance matrices assumption is violated. Accordingly, this study employed Pillai’s trace, which yielded a significant result (*p* < 0.001).

Thus, a Welch’s ANOVA [43,44] test was performed on the five factor scores (see Table 1 for details). The results indicate significant differences in scores across each co-occurrence pattern, dimension, factor, or network. It can be inferred that Dim.1 “Narrative vs. Rational Discourse” has the highest variation value, followed by Dim.4 “Uncertainty,” which reflects the two most significant differences between the academic and entertainment registers.

Multiple comparison post hoc analyses were conducted to identify pairwise differences at the register level. Prior to these analyses, we verified the equal variance assumption for the error terms of each factor’s scores. The results clearly indicated that the equal variance assumptions were violated, as evidenced by Levene’s test for equality of error variances, which yielded *p*-values as low as 0.000. Consequently, Dunnett’s T3 method was selected for multiple comparisons, as [45] demonstrated its effectiveness in controlling Type I error when the homogeneity of variance assumption is violated, thereby offering a more conservative approach.

**Figure 2:**
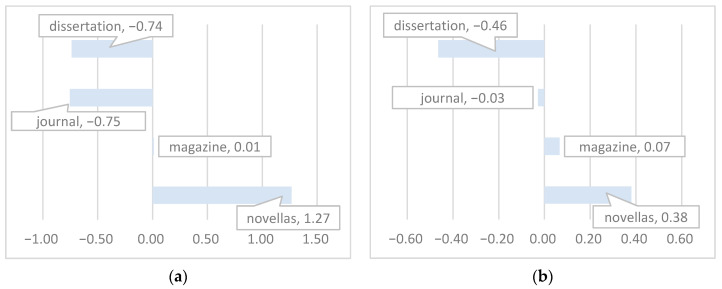

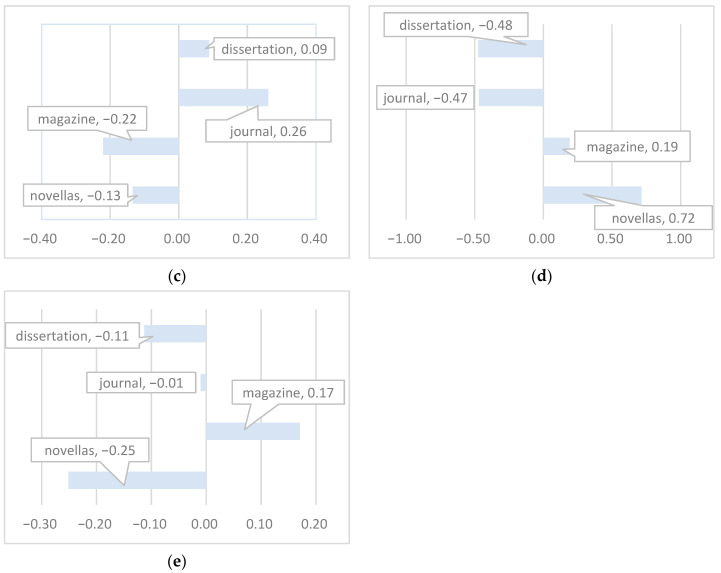
Mean scores of four categories in each dimensions: (**a**) mean scores of four categories in Dim.1 Narrative vs. Rational; (**b**) mean scores of four categories in Dim.2 Modification; (**c**) mean scores of four categories in Dim.3 Reference; (**d**) mean scores of four categories in Dim.4 Uncertainty; (**e**) mean scores of four categories in Dim.5 Prudence.

Dunnett’s T3 multiple comparisons of mean scores for each factor (refer to Appendix C for details) revealed the following significant findings: (1) there are notable differences in scores for all five factors between academic and entertainment Chinese texts; (2) research dissertations show differing scores from journal articles for both the first and fifth factors; and (3) novellas exhibit significant score differences compared to magazine articles across all five factors. These findings are illustrated in greater detail in Figure 2.

**Figure 3:**
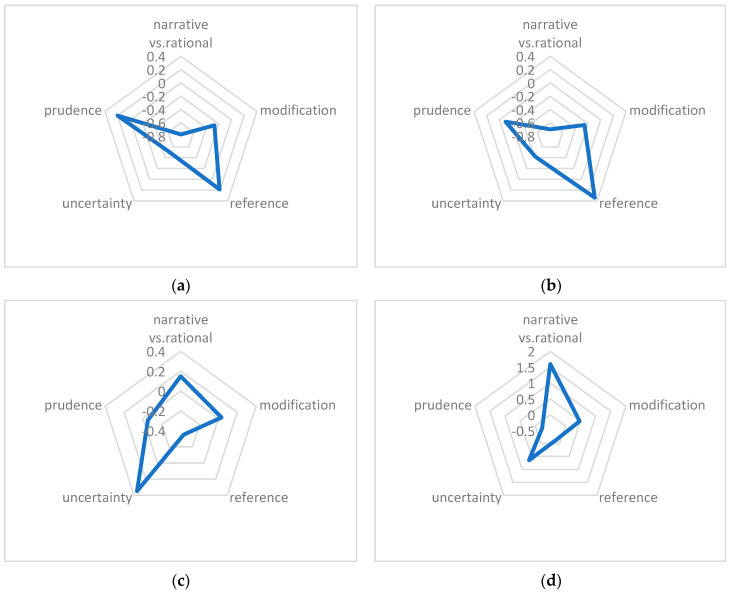
Dimension scores of each category in all dimensions: (**a**) dimension scores of journal papers; (**b**) dimension scores of research dissertations; (**c**) dimension scores of magazine articles; (**d**) dimension scores of novellas.

To better conceive the variations among the written texts under investigation, we can refer to the radar charts presented in Figure 3, which visually illustrate the differences across key dimensions and provide a comparative overview of the distinct patterns in the data.

In summary, Figure 3 offers a clear visual representation that demonstrates significant consistency between journal papers and research dissertations across several dimensions, indicating a high degree of similarity. In contrast, magazine articles and novellas show considerable differences across multiple dimensions.

### 3.3. Entropy Calculation Results

This section presents the results of entropy calculations, analyzing the differences in character-level entropy, word-level entropy, and sentence-length entropy across various text types, specifically academic and entertainment texts. The analysis aims to demonstrate how entropy values reflect the complexity of language and the flow of information within these two categories.

A Python program (https://www.python.org/) was developed to calculate the entropy values for all texts in the corpus, generating three distinct types of entropy results. The following subsections detail the outcomes for each entropy type, emphasizing how these values illustrate the complexity and variability of language use across different text types.

#### 3.3.1. Character-Level Entropy Results

As shown in Table 2, character-level entropy is higher in entertainment texts (magazines and novellas) than in academic texts (journals and dissertations). The mean entropy for academic texts is 8.169, whereas entertainment texts show a mean entropy of 8.632. This indicates that entertainment texts demonstrate greater variability in character usage, suggesting a more complex and less structured language. In contrast, academic texts are generally more controlled and predictable in their character selection.

Variance analysis (*F* = 416.292, *p* = 0.000) confirms significant differences among text categories, demonstrating a large effect size (*η*^2^ = 0.556). Post hoc tests presented in Table 3 indicate statistically significant pairwise comparisons between categories (*p* = 0.000). Additionally, a *t*-test (*t* = −28.144, *p* = 0.000) reveals a significant difference in entropy between academic and entertainment texts, with entertainment texts exhibiting higher entropy.

Thus, the higher entropy values in entertainment texts indicate a greater complexity and unpredictability in character usage, reflecting the more dynamic and varied linguistic structures found in these texts compared to the more structured academic ones.

#### 3.3.2. Word-Level Entropy Results

As illustrated in Table 4, the mean word-level entropy for academic texts is 8.473, with journals (8.268) showing slightly lower than dissertations (8.782). In contrast, entertainment texts exhibit higher (8.997) with magazines (8.993) and novellas (9.001). Overall, the former demonstrates greater variability and complexity in word usage compared to the latter, emphasizing the richness and flexibility of language in non-academic contexts.

Variance analysis (*F* = 323.817, *p* = 0.000) confirms significant differences among text categories, showing a large effect size (*η*^2^ = 0.494). Post hoc tests in Table 5 indicate significant differences among all groups, except for magazines and novellas (*p* = 0.999). Furthermore, a *t*-test (*t* = −22.539, *p* = 0.000) shows entertainment texts have higher entropy values.

In conclusion, word-level entropy is significantly higher in entertainment texts, indicating greater variability and complexity in word choice, a characteristic of the diverse linguistic structures found in non-academic texts. Moreover, magazines and novellas do not show significant differences, suggesting a high degree of similarity in word usage between the two categories.

#### 3.3.3. Sentence-Length Entropy Results

Table 6 presents the results for sentence-length entropy. Academic texts exhibit a higher average sentence-length entropy of 6.327, with journals showing a mean of 6.121 and dissertations indicating a mean of 6.635. In contrast, entertainment texts display lower sentence-length entropy, with magazines averaging 6.029 and novellas averaging 5.880, resulting in a total entertainment average of 5.969.

Variance analysis (*F* = 295.970, *p* = 0.000) confirms significant differences across text categories and indicates a large effect size (*η*^2^ = 0.471). The post hoc tests presented in Table 7 reveal significant differences among all groups, with *p*-values of 0.000 for each comparison. A *t*-test (*t* = 17.112, *p* = 0.000) confirms that academic texts exhibit significantly higher entropy of sentence length compared to entertainment texts, highlighting a contrasting phenomenon when viewed alongside the previously discussed entropy results of the other two types.

The lower entropy values of sentence length in entertainment texts suggest more consistent sentence length structures, indicating a straightforward and more accessible narrative style. In contrast, the higher entropy observed in academic texts reflects a greater variety and complexity in sentence structures, aligning with the formal, analytical, and detailed nature of academic writing. For instance, academic texts typically consist of various chapters and sections, each serving a significant function; therefore, the sentence lengths in different components tend to vary. Conversely, novellas and magazine articles generally lack such distinct functional sections, resulting in more uniform sentence lengths.

## 4. Discussion

In Section 3.1 and Section 3.2, we revealed five dimensions for summarizing language variations across the same four registers through a multi-dimensional analysis of 97 linguistic features. The dimensions emerging from this study are (i) narrative versus rational discourse, (ii) modification, (iii) reference, (iv) uncertainty, and (v) prudence. The first dimension, quite similar to the universal discourse ‘narrative versus non-narrative discourse’ proposed by Biber [17], provides preliminary evidence that such a universal dimension of language variation also applies to Chinese written texts, at least when examining the language uses for registers in academic and entertainment contexts. The study found that the two academic registers differed significantly from the two entertainment registers in four out of the five dimensions.

Language in research dissertations and journal papers is found to be much more similar to one another, except for the first and fifth dimensions. More specifically, language in journal papers generally appears to be more rational and prudent than in research dissertations. Given that the vast majority of journal papers are authored by academic scholars while research dissertations are completed by graduate students, this finding seems highly reasonable. Based on this finding, dissertation supervisors or instructors of thesis writing should communicate more effectively with graduate students about language use approaches to enhance the level of academese in dissertations. More studies with careful design in this area are needed to determine whether this is a unique case in Chinese or a more prevalent issue in other languages as well.

Meanwhile, novellas and magazine articles differ in their language usage across each of the five dimensions, although both serve as written registers for entertainment purposes and are clearly distinct from academic registers. This indicates that MDA studies of written Chinese registers for non-academic purposes are very promising and warrant more attention and effort.

The above findings are also consistent with the results of the entropy value calculations, which can measure language uncertainty—a topic considered in multidisciplinary research [46]—especially as an indicator of Dim.4 “Uncertainty,” with its importance second only to Dim.1. In Section 3.3, we calculated three types of entropy values for four Chinese registers across 1000 texts. Our findings indicate that the average entropy values at both the character and word levels for entertainment texts, such as magazine articles and novellas, are significantly higher than those for academic texts, including journal papers and research dissertations. This is completely consistent with the distribution of mean scores of Dim.4 across different register texts, showing that entertainment has higher uncertainty than academic in character and word use.

We were also surprised to find that the distribution of uncertainty shows certain similarities with the distributions of Dim.1 and Dim.2, indicating that entertainment texts place more emphasis on “narrative discourse” and “modification, “which are associated with higher uncertainty in language due to various adjectives, stative verbs, and other components. In contrast, academic texts focus more on “rational discourse” and a lack of “modification,” using more fixed and less varied vocabulary, which in turn reduces the uncertainty in linguistic expression. Additionally, we noted significant differences in entropy values between any two types of texts based on all three measures, with the exception of novellas and magazine articles, which do not show a significant difference in word-level entropy. This indicates that the entertainment register has powerful functions in communication, causing novellas and magazines to exhibit similarities in certain aspects.

The above findings resemble those of previous research. For instance, Chen et al. [47] also explored the entropies of various text types, identifying a similar distribution pattern of entropy for word forms and parts of speech (POS) in both Chinese and English. Their research found that news texts exhibited higher relative entropy of word forms, while academic texts demonstrated lower entropy values, suggesting significant differences in the syntactic structures of narrative versus expository text types. Our study similarly finds that entertainment texts show higher entropy values than academic texts, particularly at the character and word levels, which may reflect the richer linguistic features and expressions characteristic of entertainment texts.

However, concerning sentence length, the average entropy of entertainment texts is significantly lower than that of academic texts, which is similar to the distribution of mean scores of the texts in Dim.3 “reference.” In general, academic texts require a rigorous process of argumentation, often involving extensive citations to ensure the credibility and comprehensiveness of the arguments, which increases the uncertainty of sentence length. On the other hand, entertainment texts focus on capturing the reader’s interest and providing entertainment, using simple, direct language with an emphasis on emotional resonance. To reach a broad audience, entertainment texts typically avoid excessive theoretical citations and complex sentence structures.

Yu and Jiang [48] focused on the colligational diversity of lexical and grammatical words in Chinese, using entropy values to explore how the collocational behavior of words changes through grammaticalization. In their research, an increase in entropy values indicates greater variation and semantic bleaching associated with grammaticalization. Although our study does not directly address the process of grammaticalization, the observed differences at the sentence-length level suggest that entertainment and academic texts diverge in their linguistic structures. Academic texts, which are often more rigidly structured and uniform in sentence length, exhibit lower entropy values. This structural linguistic feature may reflect the trends they describe, where grammaticalization leads to increased syntactic regularity and constraint, while entertainment texts display more linguistic variation.

In this study, it is also shown that three types of entropy values in Chinese texts can serve as linguistic features to distinguish between academic and entertainment registers, as well as their sub-registers. This supports previous studies suggesting that entropy values are influenced by both language and genre, demonstrating strong sensitivity to these distinctions [36,37].

Through MDA analysis of a large-scale corpus, we empirically confirmed certain claims in previous literature regarding the differences between academic and non-academic Chinese. More specifically, about two-thirds of the 97 linguistic features mentioned in previous works as marked features of academic or formal Chinese were found to be meaningful in distinguishing between academic and entertainment Chinese texts. For instance, the 73 linguistic features finally proven to be meaningful as distinguishing features between academic and nonacademic texts in Chinese include ‘specific classical Chinese words’, ‘oral words’, and ‘er-words’, as proposed by Li [40] and Wu [49], as well as indicators of degree of formality, as argued by Feng et al. [39].

Over 30 linguistic features are not included in any of the five dimensions. One possible reason is that the academic texts analyzed were all from the fields of humanities and social sciences, excluding those from STEM fields. Therefore, features like ‘concrete terms of science and technology’ were not found to be meaningful in identifying language variations across the four registers. For most of the other features, the primary reason is simply the lack of sufficient evidence for their significant role in distinguishing between academic and entertainment registers. For example, the frequent use of compound sentences has long been recognized as a characteristic of academic Chinese. However, eight out of the ten compound sentences were actually more frequently used in novellas or magazine articles than in the two academic registers. Such findings highlight the importance of an empirical investigation of language use in written Chinese texts, as this study demonstrates.

More specifically, this study revealed significant differences between academic dissertations and journal papers in terms of Dim.1 “Narrative vs. Rational”, Dim.5 “Prudence”, and three kinds of entropy values. While both dissertations and journal papers in Dim.1 exhibit a rational function, the former demonstrates a notably weaker degree of rationality and tends more toward a narrative style. Additionally, dissertations show a stronger prudence function compared to journal papers, and their entropy values at the character, word length, and sentence length levels are also higher.

From the perspective of network implications, this phenomenon could be interpreted as reflecting the influence of social networks on language functions. Register is, on that account, intra-individual functional linguistic variation in a specific social setting [50]. Research papers published in academic journals typically address cutting-edge topics in academic fields and are written by senior scholars who require a more rational style of expression. In contrast, dissertations are written by graduate students, who are less experienced in research and whose content tends to be less innovative, which may lead to a greater reliance on a narrative style. Furthermore, due to the limited duration of academic training, students may display greater prudence and uncertainty [51] in their use of academic vocabulary and sentence structures in writing.

This distinction underscores the authoritative role that senior scholars or teachers occupy within academic networks, where rational discourse serves as a fundamental norm. The proficient use of such discourse by scholars not only reinforces their position within the network but also contributes to the maintenance of hierarchical structures. This hierarchical dynamic can limit students’ roles, positioning them as passive receivers of information rather than active, equal participants in collaborative academic networks. Consequently, it becomes crucial to examine the social function of rational discourse, particularly its role in shaping students’ visibility and influence within these networks.

The ability to effectively engage in rational discourse may enable students to navigate and assert themselves within these networks, potentially enhancing their academic capital and collaborative opportunities. In certain contexts, such as interdisciplinary teams, students’ narrative skills may offer distinct advantages, allowing for more flexible interaction and knowledge exchange across disciplinary boundaries. Therefore, we advocate for the use of social network analysis tools (e.g., Gephi [52]) to map and visualize how discourse differences between teachers and students influence collaboration patterns and overall network dynamics. These approaches would provide a deeper understanding of how discourse shapes power relations and collaboration flows within academic settings, offering valuable insights into the complex social networks that underpin academic collaboration.

## 5. Conclusions

This study underscores the crucial role of language functions in shaping linguistic features within academic and entertainment registers. By employing multi-dimensional analysis (MDA) and entropy calculations on a large-scale Chinese corpus, we have identified five key language functions that account for a significant portion of the variation in language use across these registers. These functions demonstrate how specific linguistic features co-occur to create functional patterns that affect communication in various contexts.

The findings emphasize the intricate relationship between linguistic features and language functions, offering a novel theoretical framework for understanding how language adapts to different communicative environments. By investigating how these functions shape language in both academic and entertainment contexts, this study provides valuable insights into the evolution of social networks and the dynamics of communication across varied settings.

In conclusion, the integration of multi-dimensional analysis with entropy measures facilitates a deeper understanding of how language functions influence linguistic variation. This research contributes to broader theoretical discussions on language use and communication while offering practical implications for understanding the dynamics of social interaction in different registers. The limitations of this study highlight several areas for future improvement. First, there is a lack of a stronger theoretical synthesis between functional linguistics and information theory, which would provide a more comprehensive framework for understanding language functions in social networks. Second, due to the violation of homogeneity, applying a generalized linear model may offer better results and more robust insights in data analysis. Third, this paper lacks actual modeling or data from real-world networks, which limits the ability to directly test the theoretical concepts discussed.

In terms of future work, we aim to refine the analytical framework to enhance its applicability across diverse communicative contexts, thereby expanding its potential to examine the complexities of language functions within social networks. While this study focused primarily on linguistic features, future research could explore actual co-authorship networks to examine whether teachers’ use of rational discourse correlates with higher brokerage centrality or if students’ narrative styles predict bridging roles in less formalized networks. Additionally, it will be crucial to investigate a theoretical synthesis between functional linguistics and information theory to create a more integrated approach to analyzing language in social structures.

## Figures and Tables

**Table 1:** The univariate test result for dimension score.

Dimension	*df* 1	*df* 2	*F*	Sig.	R^2^	Variation Accounted
Dim.1 Narrative vs. Rational Discourse	3	976	1302.128	0.000 ***	0.800	80%
Dim.2 Modification	3	976	34.283	0.000 ***	0.095	9.5%
Dim.3 Reference	3	976	31.392	0.000 ***	0.088	8.8%
Dim.4 Uncertainty	3	976	108.890	0.000 ***	0.251	25.1%
Dim.5 Prudence	3	976	11.259	0.000 ***	0.033	3.3%

*** *p* < 0.001.

**Table 2:** Descriptive statistics of character-level entropy.

	Category	Mean	SD	Case
Academic	journal	8.062	0.288	300
	dissertation	8.328	0.263	200
	total	8.169	0.307	500
Entertainment	magazine	8.717	0.154	300
	novella	8.506	0.204	200
	total	8.632	0.204	500
Grand Total		8.401	0.349	1000

**Table 3:** Post hoc multiple comparison *p*-values of character-level entropy.

Category 1	Category 2	Sig.	Category 1	Category 2	Sig.
journal	dissertation	0.000 ***	magazine	journal	0.000 ***
	magazine	0.000 ***		dissertation	0.000 ***
	novella	0.000 ***		novella	0.000 ***
dissertation	journal	0.000 ***	novella	journal	0.000 ***
	magazine	0.000 ***		dissertation	0.000 ***
	novella	0.000 ***		magazine	0.000 ***

*** *p* < 0.001.

**Table 4:** Descriptive statistics of word-level entropy.

	Category	Mean	SD	Case
Academic	journal	8.268	0.413	300
	dissertation	8.782	0.353	200
	total	8.473	0.464	500
Entertainment	magazine	8.993	0.189	300
	novella	9.001	0.285	200
	total	8.997	0.232	500
Grand Total		8.735	0.451	1000

**Table 5:** Post hoc multiple comparison *p*-values of word-level entropy.

Category 1	Category 2	Sig.	Category 1	Category 2	Sig.
journal	dissertation	0.000 ***	magazine	journal	0.000 ***
	magazine	0.000 ***		dissertation	0.000 ***
	novella	0.000 ***		novella	0.999
dissertation	journal	0.000 ***	novella	journal	0.000 ***
	magazine	0.000 ***		dissertation	0.000 ***
	novella	0.000 ***		magazine	0.999

*** *p* < 0.001.

**Table 6:** Descriptive statistics of sentence-length entropy.

	Category	Mean	SD	Case
Academic	journal	6.121	0.322	300
	dissertation	6.635	0.271	200
	total	6.327	0.394	500
Entertainment	magazine	6.029	0.144	300
	novella	5.880	0.337	200
	total	5.969	0.251	500
Grand Total		6.148	0.375	1000

**Table 7:** Post hoc multiple comparison *p*-values of sentence-length entropy.

Category 1	Category 2	Sig.	Category 1	Category 2	Sig.
journal	dissertation	0.000 ***	magazine	journal	0.000 ***
	magazine	0.000 ***		dissertation	0.000 ***
	novella	0.000 ***		novella	0.000 ***
dissertation	journal	0.000 ***	novella	journal	0.000 ***
	magazine	0.000 ***		dissertation	0.000 ***
	novella	0.000 ***		magazine	0.000 ***

*** *p* < 0.001.

## Data Availability

The data used to support the findings of this paper are available from the corresponding author upon reasonable request.

## Abbreviations

The following abbreviations are used in this manuscript:

MDA	multi-dimensional analysis
Dim	dimension
MANOVA	multivariate analysis of variance
ANOVA	analysis of variance
EAP	English for Academic Purposes
STEM	science, technology, engineering, and mathematics

## Appendix A

The number of asterisk signs (*) indicates how many dimensions/co-occurrence patterns/factors a linguistic feature belongs to. A linguistic feature without any asterisk sign means it was not a constituent of any of the six dimensions derived in this study.

**Table A1:** The 97 linguistic features under analysis in this study.

Categories	Linguistic Features
Nouns & Special Noun Genus	1. Noun: most commonly used ^b^*; 2. Noun: moderately commonly used; 3. Noun: rarely used; 4. Abstract noun *; 5. Stance noun *; 6. Mental noun *; 7. University subject classification *; 8. Personal noun; 9. Collective noun; 10. Figurative noun; 11. Metric and measure **
Verbs & Special Verb Genus	12. Verb: most commonly used ^b^; 13. Verb: moderately commonly used; 14. Verb: rarely used; 15. Action verb *; 16. Causative verb; 17. Existing verb; 18. Mental verb *; 19. Indicative verb; 20. Communicative verb *; 21. Speculative verb *; 22. Directional verb ^a^*; 23. Adverbial verb ^a^ (e.g., 带头‘leading’, 合力‘working together’); 24. Light verb ^a^** (e.g., 进行‘progressing’, 给予‘giving’)
Adjectives & Special Adj. Genus	25. Adjective: most commonly used ^b^; 26. Adjective: moderately commonly used *; 27. Adjective: rarely used *; 28. Configurational adjective; 29. State word ^a^*; 30. Distinguishing word ^a^*
Numerals & Quantifiers	31. Numeral ^a^*; 32. Quantifier ^a^*
Pronouns	33. 我‘First person pronoun: I’ ^d^*; 34. 我们‘First person pronoun: we’ ^d^*; 35. Less frequently used first person pronoun (e.g., 俺, 本人) ^d^; 36. Second person pronoun *; 37. Third person pronoun *; 38. 它‘Inanimate pronoun: it’ *; 39.们‘Common noun + plural’ ^d^; 40. Demonstrative pronoun; 41. Indefinite pronoun *
Adverbs & Special Adv. Genus	42. Adverb: most commonly used *; 43. Adverb: moderately commonly used *; 44. Adverb: rarely used *; 45. Obligatory adverb *; 46. Adverb: possibility *; 47. Adverb: attitude *
Preposition	48. Preposition
Auxiliaries	49. 的‘Possessive affix: de’ ^a^*; 50. 地‘Adverbializer: de’ ^a^; 51. 得‘Resultative complementizer: de’ ^a^*; 52. 等/等等‘Analogous particle: deng/dengdeng’ ^a^*; 53. Equality adverb ^a^*
Other Lexical Genera	54. Modal particle ^a^*; 55. Sequential word ^d^*; 56. Phrasal coordination *; 57. Spoken word ^d^*; 58. Er-hua word ^d^*(e.g., 小孩儿‘kid’); 59. Monosyllabic word used in disyllabic template ^d^* (e.g., 返京‘return to Beijing’); 60. Couple disyllabic word ^d^* (e.g., 禁止‘prohibition’, 安装‘installation’); 61. Classical word ^d^*; 62. Degree adverb ^a^*; 63. Adverbial hedge *
Noun Forms	64. Nominalization; 65. Gerundive functional word ^a^*
State Forms	66. 是‘Copular verb be, used as the main verb’ ^a^* (e.g., 尤其是‘especially be’, 无疑是‘undoubtedly be’)
Temporal & Aspect Markers	67. Progressive construal * (e.g., 正看‘looking’, 玩耍中‘playing’); 68. Past temporal adverb * (e.g., 曾听‘heard that’); 69. 着‘Durative aspect: zhe’ ^a^*; 70. 了‘Past aspect: le’ ^a^*; 71. 过‘Experiential aspect: guo’ ^a^*
Modal Verbs	72. Obligatory modal; 73. Possibility modal
Adverbials of Place/Time	74. Adverb of time *; 75. Locative word *
Contractions	76. Abbreviation
Negatives	77. Negative word ^a^*
Independent Words	78. Interjection ^a^*; 79. Onomatopoeia ^a^*; 80. Parenthesis ^d^*
Questions	81. Interrogative Sentence *
Passives	82. Passive construal; 83. Passive sentence without doer; 84. 把‘Preposition ba sentence’ ^a^*
Compound Sentences	85. Parallel compound sentence ^c^*; 86. Successive compound sentence ^c^*; 87. Explanatory compound sentence ^c^*; 88. Selective compound sentence ^c^; 89. Progressive compound sentence ^c^*; 90. Conditional compound sentence ^c^**; 91. Hypothetical compound sentence ^c^**; 92. Causal compound sentence ^c^*; 93. Purpose compound sentence^c^; 94. Turning compound sentence ^c^*
Lexical specifications	95. Type/token ratio ^e^*; 96. Average word length ^e^*; 97. Average sentence length ^e^

## Appendix B

The summary of the factorial solutions of the present study is shown below, displayed according to the linguistic features contained in each dimension, with the values in parentheses representing the loadings.

### Appendix B.1. Dim.1 Narrative vs. Rational Discourse

(positive features)

着 ‘durative aspect: zhe’ (1.15), modal particle (1.09), past temporal adverb (1.05), second person pronoun (1.01), directional verb (0.99), onomatopoeia (0.97), communicative verb (0.95), 了 ‘past aspect: le’ (0.95), 把 ‘preposition ba sentence’ (0.92), interrogative sentence (0.92), 我 ‘first person pronoun: I’ (0.89), interjection (0.88), third person pronoun (0.88), spoken word (0.87), indefinite pronoun (0.86), 得 ‘resultative complementizer: de2’ (0.86), state word (0.85), 地 ‘adverbializer: de’ (0.85), negative word (0.77), progressive construal (0.76), 过 ‘experiential aspect: guo’ (0.75), successive compound sentence (0.75), equality adverb (0.74), obligatory adverb (0.73), speculative verb (0.70), parenthesis (0.69), adverb of time (0.68), adverb: attitude (0.64), less frequently used first person pronoun (0.61), adverb: most commonly used (0.61), conditional compound sentence (0.60), hypothetical compound sentence (0.55), mental verb (0.55), numeral (0.52), degree adverb (0.50), quantifier (0.47), locative word (0.42), demonstrative pronoun (0.39), action verb (0.39), possibility modal (0.38)

(negative features)

couple disyllabic word (−0.71), gerundive functional word (−0.70), 等/等等 ‘analogous: deng/deng deng’ (−0.65), phrasal coordination (−0.60), average word length (−0.59), noun: most commonly used (−0.56), abstract noun (−0.52), type/token ratio (−0.48), light verb (−0.47), average sentence length (−0.46), distinguishing word (−0.41), adverbial verb (−0.39), metric & measure (−0.38), purpose compound sentence (−0.37), 的‘possessive affix: de’ (−0.36), explanatory compound sentence (−0.35)

### Appendix B.2. Dim.2 Modification

adverb: moderately commonly used (0.73), monosyllabic word used in disyllabic template (0.56), adjective: moderately commonly used (0.50), adverb: rarely used (0.49), adjective: rarely used (0.38)

### Appendix B.3. Dim.3 Reference

mental nouns (0.70), 它 ‘inanimate pronoun: it’ (0.61), 的 ‘possessive affix: de’ (0.58), 是 ‘copular verb be used as the main verb’ (0.52), 我们 ‘first person pronoun: we’ (0.45), university subject classification (0.42)

### Appendix B.4. Dim.4 Uncertainty

adverbial hedge (1.03), adverb: possibility (0.74)

### Appendix B.5. Dim.5 Prudence

numeral (0.69), sequential word (0.60), classical word (0.59), metric & measure (0.55)

## Appendix C

The results of multiple comparisons of the means of dimension scores are shown below, displayed in the order of the dimensions.

**Table A2:** *P*-Values for the Dunnett’s T3 multiple comparisons of means of Dim.1.

Category 1	Category 2	Sig.	Category 1	Category 2	Sig.
journal	dissertation	0.033 *	magazine	journal	0.000 ***
	magazine	0.000 ***		dissertation	0.000 ***
	novella	0.000 ***		novella	0.000 ***
dissertation	journal	0.033 *	novella	journal	0.000 ***
	magazine	0.000 ***		dissertation	0.000 ***
	novella	0.000 ***		magazine	0.000 ***

* *p* < 0.05; *** *p* < 0.001.

**Table A3:** *P*-Values for the Dunnett’s T3 multiple comparisons of means of Dim.2.

Category 1	Category 2	Sig.	Category 1	Category 2	Sig.
journal	dissertation	1.000	magazine	journal	0.000 ***
	magazine	0.000 ***		dissertation	0.003 **
	novella	0.000 ***		novella	0.000 ***
dissertation	journal	1.000	novella	journal	0.000 ***
	magazine	0.003 **		dissertation	0.000 ***
	novella	0.000 ***		magazine	0.000 ***

** *p* < 0.01; *** *p* < 0.001.

**Table A4:** *P*-Values for the Dunnett’s T3 multiple comparisons of means of Dim.3.

Category 1	Category 2	Sig.	Category 1	Category 2	Sig.
journal	dissertation	0.535	magazine	journal	0.000 ***
	magazine	0.000 ***		dissertation	0.000 ***
	novella	0.000 ***		novella	0.006 **
dissertation	journal	0.535	novella	journal	0.000 ***
	magazine	0.000 ***		dissertation	0.000 ***
	novella	0.000 ***		magazine	0.006 **

** *p* < 0.01; *** *p* < 0.001.

**Table A5:** *P*-Values for the Dunnett’s T3 Multiple Comparisons of Means of Dim.4.

Category 1	Category 2	Sig.	Category 1	Category 2	Sig.
journal	dissertation	0.823	magazine	journal	0.000 ***
	magazine	0.000 ***		dissertation	0.000 ***
	novella	0.000 ***		novella	0.003 **
dissertation	journal	0.823	novella	journal	0.000 ***
	magazine	0.000 ***		dissertation	0.000 ***
	novella	0.000 ***		magazine	0.003 **

** *p* < 0.01; *** *p* < 0.001.

**Table A6:** *P*-Values for the Dunnett’s T3 multiple comparisons of means of Dim.5.

Category 1	Category 2	Sig.	Category 1	Category 2	Sig.
journal	dissertation	0.007 **	magazine	journal	0.011 *
	magazine	0.011 *		dissertation	0.978
	novella	0.000 ***		novella	0.001 **
dissertation	journal	0.007 **	novella	journal	0.000 ***
	magazine	0.978		dissertation	0.173
	novella	0.173		magazine	0.001 **

* *p* < 0.05; ** *p* < 0.01; *** *p* < 0.001.

## References

[1] Allen M.L., Elliott M.N., Fuligni A.J., Morales L.S., Hambarsoomian K., Schuster M.A. (2008). The Relationship Between Spanish Language Use and Substance Use Behaviors Among Latino Youth: A Social Network Approach. J. Adolesc. Health.

[2] Scholand A.J., Tausczik Y.R., Pennebaker J.W. Social Language Network Analysis. Proceedings of the ACM Conference on Computer Supported Cooperative Work.

[3] McManus K. (2019). Relationships between social networks and language development during study abroad. Lang. Cult. Curric..

[4] Ke J., Gong T., Wang W.S.Y. (2008). Language change and social networks. Commun. Comput. Phys..

[5] Sharma D., Dodsworth R. (2020). Language Variation and Social Networks. Annu. Rev. Linguist..

[6] South T., Smart B., Roughan M., Mitchell L. (2022). Information flow estimation: A study of news on Twitter. Online Soc. Netw. Media.

[7] Francis T., Davidson M., Senese L., Jeffs L., Yousefi-Nooraie R., Ouimet M., Rac V., Trbovich P. (2024). Exploring the use of social network analysis methods in process improvement within healthcare organizations: A scoping review. BMC Health Serv. Res..

[8] Bryden J., Funk S., Jansen V.A.A. (2013). Word usage mirrors community structure in the online social network Twitter. EPJ Data Sci..

[9] Biber D., Conrad S. (2009). Register, Genre, and Style.

[10] Biber D., Conrad S. (2019). Register, Genre, and Style.

[11] Biber D. (2006). University Language: A Corpus-Based Study of Spoken and Written Registers.

[12] Nesi H., Gardner S. (2012). Genres Across the Disciplines: Student Writing in Higher Education.

[13] Zhu X. (2015). A Multi-Dimensional Approach to Register Variation in Mandarin Chinese. Master’s Thesis.

[14] Biber D. (1985). Investigating Macroscopic Textual Variation Through Multifeature Multidimensional Analyses. Linguistics.

[15] Biber D. (1986). Spoken and Written Textual Dimensions in English—Resolving the Contradictory Findings. Language.

[16] Biber D. (1988). Variation Across Speech and Writing.

[17] Biber D. (2014). Using multi-dimensional analysis to explore cross-linguistic universals of register variation. Lang. Contrast.

[18] Biber D., Gray B., Staples S. (2016). Predicting Patterns of Grammatical Complexity Across Language Exam Task Types and Proficiency Levels. Appl. Linguist..

[19] Hardy J.A., Friginal E. (2016). Genre variation in student writing: A multi-dimensional analysis. J. Engl. Acad. Purp..

[20] Friginal E., Li M., Weigle S.C. (2014). Revisiting multiple profiles of learner compositions: A comparison of highly rated NS and NNS essays. J. Second. Lang. Writ..

[21] Weigle S.C., Friginal E. (2015). Linguistic dimensions of impromptu test essays compared with successful student disciplinary writing: Effects of language background, topic, and L2 proficiency. J. Engl. Acad. Purp..

[22] Crosthwaite P. (2016). A longitudinal multidimensional analysis of EAP writing: Determining EAP course effectiveness. J. Engl. Acad. Purp..

[23] Thompson P., Hunston S., Murakami A., Vajn D. (2017). Multi-Dimensional Analysis, text constellations, and interdisciplinary discourse. Int. J. Corpus Linguist..

[24] Egbert J. (2015). Publication type and discipline variation in published academic writing Investigating statistical interaction in corpus data. Int. J. Corpus Linguist..

[25] Monaco L.M. (2016). Was late Modern English scientific writing impersonal? Comparing Philosophy and Life Sciences texts from the Coruna Corpus. Int. J. Corpus Linguist..

[26] Gray B. (2013). More than discipline: Uncovering multi-dimensional patterns of variation in academic research articles. Corpora.

[27] Hardy J.A., Römer U. (2013). Revealing disciplinary variation in student writing: A multi-dimensional analysis of the Michigan Corpus of Upper-level Student Papers (MICUSP). Corpora.

[28] Egbert J. (2012). Style in nineteenth century fiction: A Multi-Dimensional analysis. Sci. Study Lit..

[29] Grieve J., Biber D., Friginal E., Nekrasova T. (2010). Variation Among Blogs: A Multi-dimensional Analysis. Genres on the Web.

[30] Egbert J., Biber D. (2016). Do all roads lead to Rome?: Modeling register variation with factor analysis and discriminant analysis. Corpus Linguist. Linguist. Theory.

[31] Sardinha T.B. (2018). Dimensions of variation across Internet registers. Int. J. Corpus Linguist..

[32] Berber Sardinha T., Veirano Pinto M. (2019). Dimensions of variation across American television registers. Int. J. Corpus Linguist..

[33] Shannon C. (1948). A mathematical theory of communication. Bell Syst. Tech. J..

[34] Stanisz T., Drozdz S., Kwapien J. (2024). Complex systems approach to natural language. Phys. Rep.-Rev. Sect. Phys. Lett..

[35] Christian B., Dimitrios A., Michael C., Ramon F.I.C. (2017). The Entropy of Words—Learnability and Expressivity across More than 1000 Languages. Entropy.

[36] Papadimitriou C., Karamanos K., Diakonos F.K., Constantoudis V., Papageorgiou H. (2010). Entropy analysis of natural language written texts. Phys. A Stat. Mech. Its Appl..

[37] Kalimeri M., Constantoudis V., Papadimitriou K., Karamanos K., Diakonos F.K., Papageorgiou H. (2012). Entropy analysis of word-length series of natural language texts: Effects of text language and genre. Int. J. Bifurc. Chaos.

[38] Huang B., Liao X. (2017). Xiandai Hanyu (Xiuding Liu Ban) [Modern Chinese].

[39] Feng S., Wang J., Huang M. (2008). Hanyu shumian yuti zhuangyadu de zidong celiang [An automatic feature checking algorithm for degree of formalities in written Chinese]. Yuyan Kexue [Linguist. Sci.].

[40] Li Y. (1989). Hanyu Yuti Xiucixue [Chinese Genre Rhetorics].

[41] Shannon C.E., Weaver W. (1949). The Mathematical Theory of Communication.

[42] Pillai K.C.S. (1955). Some New Test Criteria in Multivariate Analysis. Ann. Math. Stat..

[43] Delacre M., Lakens D., Leys C. (2017). Why Psychologists Should by Default Use Welch’s *t*-test Instead of Student’s *t*-test. Int. Rev. Soc. Psychol..

[44] Celik N. (2022). Welch’s ANOVA: Heteroskedastic skew-*t* error terms. Commun. Stat.-Theory Methods.

[45] Sauder D., DeMars C. (2019). An Updated Recommendation for Multiple Comparisons. Adv. Methods Pract. Psychol. Sci..

[46] Dagtas A., Sahinkarakas S. (2024). Foreign Language Learners’ Uncertainty Experiences and Uncertainty Management. J. Psycholinguist. Res..

[47] Chen R., Liu H., Altmann G. (2017). Entropy in different text types. Digit. Scholarsh. Humanit..

[48] Yu B.Y., Jiang Y. (2024). Exploring Colligation Diversity and Grammaticalization in Chinese: An Entropy-Based Approach. J. Quant. Linguist..

[49] Wu L. (2016). Xiandai Hanyu Xiucixue (Di San Ban) [Modern Chinese Rhetorics].

[50] Pescuma V., Serova D., Lukassek J., Sauermann A., Schäfer R., Adli A., Bildhauer F., Egg M., Hülk K., Ito A. (2023). Situating language register across the ages, languages, modalities, and cultural aspects: Evidence from complementary methods. Front. Psychol..

[51] Park J., Joo K.H. (2020). A Qualitative Study on the Process of Writing a Master’s Thesis for a Graduate Students in the School of Counseling. Korean J. Qual. Res. Soc. Welf..

[52] Yang J., Cheng C., Shen S., Yang S. Comparison of Complex Network Analysis Software: Citespace, SCI^2^ and Gephi. Proceedings of the 2nd IEEE International Conference on Big Data Analysis (ICBDA).

